# Aerococcus Urinae Endocarditis: A Case Report and Literature Review

**DOI:** 10.7759/cureus.29853

**Published:** 2022-10-03

**Authors:** Khalid Saeed Al-Asad, Naveed Mazhar, Shaurya Srivastava, Syed Quadri, Subhashis Mitra

**Affiliations:** 1 Internal Medicine, Michigan State University, East Lansing, USA; 2 Infectious Disease, Michigan State University, East Lansing, USA

**Keywords:** aortic insufficiency, aortic regurgitation, aortic valve perforation, aerococcus urinae, endocarditis

## Abstract

A 75-year-old male, with a past medical history of chronic kidney disease stage 3 (CKD3) and a recent diagnosis of bilateral hydronephrosis and Foley catheter placement, presented to the emergency department for fever. Blood cultures grew Aerococcus urinae. Transthoracic echo (TTE) demonstrated thickened aortic valve leaflets with perforation, multiple echo densities, and severe aortic regurgitation. The patient developed decompensated congestive heart failure and cardiogenic shock. En route to surgery for emergent aortic valve replacement, the patient lost pulse and was resuscitated. The patient was subsequently transferred to the ICU where the family decided to initiate comfort care measures. This case highlights the importance and necessity of the prompt diagnosis and treatment of infective endocarditis and makes the reader aware of uncommon and rare organisms, such as Aerococcus urinae, as potential etiologies.

## Introduction

Infective endocarditis (IE) has classically been defined as an infection of the endocardial surface of the heart, the heart valves, or an indwelling cardiac device [1]. The annual incidence of endocarditis is increasing and has approached 15 cases per 100,000-person population as of 2011 [2]. Prompt diagnosis of infective endocarditis is essential, as it remains a highly morbid disease with a mortality rate of 20% at 30 days despite the wide array of antimicrobial treatments and surgical interventions that are now available [3]. Furthermore, a delay in diagnosis and treatment can lead to additional complications such as heart failure, abscess formation, valve perforation, intracardiac fistula, formation, or the development of pericarditis. Staphylococcus aureus is the most common cause of endocarditis in the world (approximately 31% of all cases), followed by the Enterococcus family [4]. Aerococcus urinae, a gram-positive, catalase-negative, alpha-hemolytic coccus, was first reported as a causative agent of IE in 1976 [5]. Our case of Aerococcus urinae IE was complicated by aortic valve leaflet perforation leading to severe aortic regurgitation and death. This case highlights the importance and necessity of the prompt diagnosis and treatment of infective endocarditis and makes the reader aware of uncommon and rare organisms, such as Aerococcus urinae, as potential etiologies.

## Case presentation

A 75-year-old Caucasian male presented to the emergency department of Sparrow Hospital, Lansing, Michigan with complaints of fever, chills, malaise, and fatigue for three days prior to presentation. His past medical history was significant for hypertension, hyperlipidemia, gastroesophageal reflux disease, stage T1c prostate cancer (Gleason 4+3) status post-robotic-assisted laparoscopic radical prostatectomy (RALP) in 2019, and the subsequent development of bladder neck contractures requiring transurethral incisions. Approximately two weeks prior to presentation, the patient was admitted with acute kidney injury, fever, dysuria, and urinary frequency. A CT scan of the abdomen and pelvis demonstrated a moderately distended bladder with marked bilateral hydronephrosis and hydroureter. A Foley was placed for treatment of acute obstructive uropathy and the patient was treated with IV ceftriaxone 1000 mg for four days. His urine culture was negative for any bacterial growth and thus antibiotics were discontinued. Repeat renal ultrasound showed improved moderate hydronephrosis. The patient was deemed stable for discharge with a Foley catheter.

His vital signs upon presentation included a temperature of 101.9 ° F, heart rate of 118 beats per minute, respiratory rate of 17, and oxygen saturation of 98% on room air. Physical examination was remarkable for a diastolic rumble best heard at the right sternal border. A complete blood count and comprehensive metabolic panel were drawn and were remarkable for an elevated white blood cell count and an elevation in serum creatinine. Urinalysis was also suggestive of a urinary tract infection with elevated WBC. He was started on piperacillin-tazobactam for gram-negative and anaerobic coverage with urine and blood cultures being drawn. He had repeat imaging with a CT abdomen and pelvis showing improvement in prior hydronephrosis, new small pericardial effusion, and bladder wall thickening and prominence. Over the course of two days, the patient's clinical course improved. Urine cultures grew Enterococcus faecalis and Alcaligenes faecalis (Table 1) while blood cultures grew Aerococcus urinae (Table 2).

**Table 1:** Urine cultures and antibiogram

	Enterococcus faecalis	Alcaligenes faecalis
	MINIMUM INHIBITORY CONCENTRATION	MINIMUM INHIBITORY CONCENTRATION
Ampicillin	1 ug/mL Susceptible	
Cefepime		4 ug/mL Susceptible
Ceftazidime		<=2 ug/mL Susceptible
Ciprofloxacin		2 ug/mL Intermediate
Gentamicin		4 ug/mL Susceptible
Levofloxacin	<=1 ug/mL Susceptible	<=0.5 ug/mL Susceptible
Linezolid	<=1 ug/mL Susceptible	
Nitrofurantoin	<=16 ug/mL Susceptible	
Piperacillin/Tazo		<=2/4 ug/mL Susceptible
Tetracycline	<=0.5 ug/mL Susceptible	
Tobramycin		<=2 ug/mL Susceptible
Trimeth/Sulfa		<=0.5/9.5 ug/mL Susceptible
Vancomycin	2 ug/mL Susceptible	

**Table 2:** Blood cultures and antibiograms Both blood cultures grew Aerococcus urinae with the same antibiograms for both sets; to prevent redundancy, we have shown results from one.

Aerococcus urinae	Bacterial Susceptibility Panel	
Ceftriaxone	1 ug/mL	Susceptible
Levofloxacin	0.25 ug/mL	Susceptible
Meropenem	0.03 ug/mL	Susceptible
Penicillin	0.06 ug/mL	Susceptible
Vancomycin	0.5 ug/mL	Susceptible

**Figure 1:**
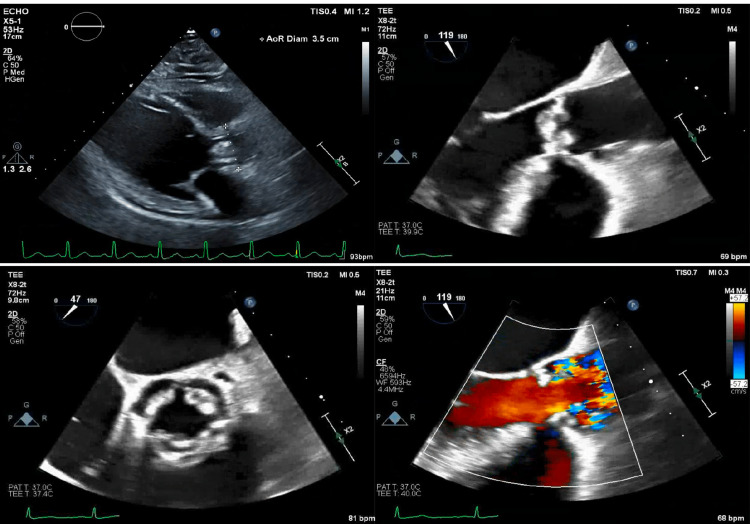
Thickened aortic valve cusps as seen on transthoracic echocardiogram (top left) and transesophageal echocardiogram (top right) Transesophageal echocardiogram displaying aortic valve echo-densities on the parasternal short access view (bottom left) and aortic regurgitation (bottom right).

TTE showed a sclerotic aortic valve with ill-defined abnormal echo densities, suggesting possible endocarditis. A transesophageal echo (TEE) showed perforation of all three aortic valve leaflets with severe regurgitation on color flow Doppler, with multiple echo densities (Figure 1).

Antibiotics were switched to IV penicillin G and IV gentamicin. Within the span of 12 hours from this result, the patient's clinical course acutely deteriorated, with the development of severe respiratory distress with tachypnea (respiratory rate 30 breaths per minute), hypotension (blood pressure was 96/35 mmHg), and sinus tachycardia (heart rate 125beats per minute). The patient was emergently taken to the operating room (OR) for open heart surgery for aortic valve replacement. En route to the OR, the patient had a cardiac arrest with pulseless electrical activity noted. Return of spontaneous circulation (ROSC) was achieved after 30 minutes, and a decision was made to abort surgery and transfer the patient to ICU. Within 30 min of arrival to the ICU unit, the patient had another cardiac arrest with pulseless electrical activity noted. The patient’s spouse made the decision to switch the patient to comfort care measures.

## Discussion

Infective endocarditis (IE) can present in days (acute) or weeks to months (subacute). Acute IE is most commonly seen with S. aureus. Subacute IE can be due to both pathogenic and non-pathogenic bacteria that usually colonize an already damaged valve. Fever in the presence of new-onset murmur, Osler's nodes, and Janeway lesions can be present [6]. Duke criteria for IE contain major and minor criteria. The major criteria are positive two separate blood cultures and an echocardiogram demonstrating evidence of vegetation. Minor criteria include fever, predisposing factors, vascular phenomena, immunological phenomena, and microbiological evidence. Patients at risk of IE are those that meet one major and minor criteria or three minor criteria [7]. Most patients receive four to six weeks of antibiotics for treatment. Surgical intervention is recommended in case of vegetation resolution being refectory to antibiotics, embolism during the first two weeks of antimicrobial therapy, vegetation after embolization, anterior mitral valve vegetation > 10 mm in size, development of new heart block, abscess formation, aortic or mitral valve dysfunction with ventricular failure, perforation along the perivalvular area, or valvular dehiscence [8].

Aerococcus urinae is a gram-positive, microaerophilic, catalase-negative, alpha-hemolytic coccus that can grow in clusters and tetrads and has the ability to form biofilms [5]. Despite it being a common cause of urinary tract infection (UTI) in males of late age (> 65), urinalysis and urine cultures can still appear negative as demonstrated not only in our case but in literature as well. Other risk factors include prostate hyperplasia, urethral strictures, and urinary tract surgery [9]. There have been case reports of infective endocarditis due to Aerococcus urinae. Subacute IE with damage to mitral and aortic valves has also been reported. In vitro, susceptibility to penicillin, cefotaxime, ceftriaxone, meropenem, tetracycline, levofloxacin, rifampin, linezolid, and vancomycin has been reported [10].

In 2020, Ludhwani et al. documented a case of a 59-year-old male with Aerococcus urinae UTI who developed aortic valve regurgitation in the setting of endocarditis. Blood cultures confirmed Aerococcus urinae bacteremia and TEE confirmed regurgitation. In this case, the patient received intravenous vancomycin and surgical aortic valve repair. Similarly, in 2019, Sous et al. documented a case of an 11-year-old boy with a history of an unrepaired ventricular septal defect. The patient had foul-smelling urine followed by fevers. Blood culture grew Aerococcus urinae. The patient initially received vancomycin then based on susceptibility results was switched to penicillin G and gentamicin. An echocardiogram showed partial closure of VSD possibly related to tricuspid valve vegetation. Subsequently, the patient developed a lobulated mycotic aneurysm of the pulmonary artery secondary to embolism from vegetation. He underwent a right lower lobectomy and completed a six-week Penicillin G course [3].

Similarly, in 2016, Kotkar et al. documented a case of a 54-year-old male who presented with myocardial infarction due to embolism of the right coronary artery. An echocardiogram revealed severe mitral and tricuspid valve regurgitation. Sternotomy revealed large vegetation on the posterior leaflet of the mitral valve with thickening of the anterior leaflet. The patient underwent an embolectomy and bypass with grafting. In this case, after the surgery, the patient was discharged on a six-week course of ampicillin [11].

In contrast to the individual cases mentioned previously, a prior comprehensive review of Aerococcus urinae infective endocarditis was completed by Yabes et al. in 2018, which compiled a tabulated report of 43 cases of Aerococcus urinae infective endocarditis [12,13]. A subsequent literature search demonstrated seven additional cases of Aerococcus urinae infective endocarditis described in Tai et al., which has brought the total number of known cases to 50 (Table 3) [14]. The average age among these 50 cases was noted to be 71 years of age, with 80% of cases noted to be in male patients. Common risk factors were most noted to be those that involved the genitourinary system (UTI, benign prostatic hyperplasia (BPH), Foley catheters, etc.) compared to the presence of valvular disease on its own. Aortic Valve involvement on its own was noted to be present among 48% of cases while sole Mitral Valve involvement comprised 44% of cases. Approximately 8% of cases rarely involved more than one valve. Surgical intervention was rare and took place in 26% of the listed cases. This is likely due to patients having an unfavorable risk profile with their underlying comorbidities, and not meeting 2014 AHA/ACC Class 1 surgical indication guidelines for Management of Patients with Valvular Heart Disease as mentioned by Yabes et al. It should be noted that the updated 2020 AHA/ACC Class 1 surgical indication guidelines for Management of Patients with Valvular Heart Disease recommend early surgical intervention in cases of infective endocarditis complicated with destructive penetrating valvular lesions even prior to completion of a full therapeutic course of antibiotics, which was not present in the 2014 guidelines [15]. Furthermore, among the 13 out of 50 cases that did receive surgical intervention, only one case died, suggesting that early surgical intervention when indicated may improve mortality outcomes. Among the antibiotic regimens used, the most common treatment course involved approximately 72% of the 50 cases receiving beta-lactam therapy with an aminoglycoside for an average duration of approximately six weeks, which resulted in approximately 78% of patients treated in this manner surviving.

**Table 3:** Tabulated case reports of Aerococcus Urinae endocarditis. AG: aminoglycoside, Van: vancomycin, BPH: benign prostatic hyperplasia, ASD: atrial septal defect, TURP: trans-urethral resection of the prostate Data from: (Ludhwani, 2020) [3], (Yabes, 2018) [12], (Tai, 2021) [14], (Feghaly, 2022) [16], (Lattanzio, 2020) [17], (Bathania, 2019) [18], (Rosborough, 2020) [19], (Martín-Guerra, 2020) [20], (Ahmed, 2021) [21], (Varughese, 2021) [22], (Khan, 2021) [23]

Case No.	Age	Sex	Risk factors	Valve	Surgery	Antibiotic regimen	Duration	Embolization	Survival
1	56	F	UTI	Aortic/Mitral Valve	Y	𝛃 Lactam/Van	2 to 4 weeks	Y	Y
2	54	M	UTI	Mitral Valve	Y	Ampicillin	6 weeks	Y	Y
3	79	F	UTI	Mitral Valve	N	𝛃 Lactam	4 weeks	Y	Y
4	72	F	UTI	Aortic Valve	N	Ampicillin	4 weeks	N	Y
5	46	M	UTI	Aortic/Mitral Valve	N	𝛃 Lactam	2 weeks	Y	N
6	80	M	UTI	Mitral Valve	Y	𝛃 Lactam/AG	4 weeks	N	Y
7	70	M	UTI	Mitral Valve	Y	𝛃 Lactam/AG	4 weeks	N	Y
8	69	M	Cystoscopy	Aortic Valve	N	𝛃 Lactam/AG	6 wks	N	Y
9	54	M	Phimosis	Mitral Valve	Y	𝛃 Lactam	6wks	Y	Y
10	43	M	Hepatitis C	Aortic Valve	N	𝛃 Lactam/AG	5 days	Y	N
11	68	M	Indwelling Catheter	Aortic Valve	Y	𝛃 Lactam/AG	6/2 wks	Y	Y
12	80	M	Malignancy	Aortic Valve	Y	𝛃 Lactam	6wks	-	Y
13	87	M	Malignancy	Mitral Valve	-	𝛃 Lactam/AG	-	N	N
14	77	M	Liver Failure	Aortic Valve	-	𝛃 Lactam/AG	-	N	Y
15	83	M	Indwelling Catheter	Mitral Valve	-	𝛃 Lactam/AG	-	N	Y
16	73	M	Suprapubic Catheter	Aortic Valve	-	𝛃 Lactam/AG	-	N	N
17	88	F	Aortic Stenosis	Aortic Valve	-	𝛃 Lactam/AG	-	N	Y
18	43	M	Indwelling Catheter	Mitral Valve	Y	𝛃 Lactam/AG	6wks	N	Y
19	77	M	BPH	Aortic Valve	N	𝛃 Lactam/Van	-	Y	N
20	68	M	BPH	Mitral Valve	N	𝛃 Lactam/AG; oral regimen	3wks; unknown	N	Y
21	75	M	Cystoscopy	Aortic Valve	Y	𝛃 Lactam/AG	6wks	Y	Y
22	89	M	TURP	Mitral Valve	N	𝛃 Lactam/AG	7days	N	N
23	81	M	UTI	Aortic Valve	N	𝛃 Lactam/AG	2wks/8days	N	N
24	91	M	Indwelling Catheter	Mitral Valve	-	𝛃 Lactam/AG	4wks/10days	N	Y
25	91	M	BPH	Mitral Valve	-	𝛃 Lactam/AG	4wks/10days	N	Y
26	89	F	-	Mitral Valve	-	𝛃 Lactam/AG	4wks/10days	N	Y
27	86	M	Urethral Stricture	Aortic Valve	-	𝛃 Lactam/AG	4wks/10days	Y	Y
28	83	M	Urethral Stricture	Mitral Valve	-	𝛃 Lactam/AG	4wks/10days	N	Y
29	80	F	-	Mitral Valve	-	𝛃 Lactam/AG	4wks/10days	N	Y
30	77	M	-	Aortic Valve	-	𝛃 Lactam/AG	4wks/10days	N	Y
31	75	M	BPH	Mitral Valve	-	𝛃 Lactam/AG	4wks/10days	N	Y
32	74	M	Suprapubic Catheter	-	-	𝛃 Lactam/AG	4wks/10days	N	Y
33	65	M	Indwelling Catheter	Mitral Valve	-	𝛃 Lactam/AG	4wks/10days	N	Y
34	53	M	Dysuria	Aortic Valve	-	𝛃 Lactam/AG	4wks/10days	N	Y
35	49	F	Intermittent catheter	Aortic Valve	-	𝛃 Lactam/AG	4wks/10days	N	Y
36	81	M	Indwelling Catheter	Mitral Valve	-	𝛃 Lactam/AG	4wks/10days	N	Y
37	74	F	Malignancy	-	-	𝛃 Lactam/AG	4wks/10days	N	Y
38	42	M	None	Aortic Valve	Y	𝛃 Lactam/AG	6wks	Y	Y
39	49	M	-	Aortic Valve	-	𝛃 Lactam/AG	-	N	Y
40	54	M	Urethral Stricture	Tricuspid/Aortic Valve	N	𝛃 Lactam/AG	-	N	N
41	69	M	Malignancy	Aortic Valve	Y	𝛃 Lactam	12wks	N	Y
42	62	M	BPH	Mitral/Aortic Valve	Y	𝛃 Lactam/Rif, AG	6wks, AG 2wks	-	Y
43	81	M	BPH	Mitral Valve	N	𝛃 Lactam/AG	6wks	N	Y
44	78	M	Indwelling Catheter	Aortic Valve	N	𝛃 Lactam	-	N	N
45	87	M	BPH	Mitral Valve	N	-	-	N	N
46	78	F	Ureteral Stent	Aortic Valve	N	𝛃 Lactam/Van	2wks	N	N
47	48	M	ASD	Mitral Valve	N	𝛃 Lactam/AG	-	Y	Y
48	79	F	UTI	Aortic Valve	N	𝛃 Lactam	6wks	Y	Y
49	78	M	Aortic Stenosis	Aortic Valve	N	𝛃 Lactam/AG	10 days	-	N
50	74	M	BPH	Mitral Valve	Y	𝛃 Lactam/AG	4wks	Y	N
51	79	M	-	Aortic Valve	N	Van	6wks	N	N
52	67	M	UTI/Bioprosthetic valve	Aortic Valve	Y	𝛃 Lactam	6wks	N	Y
53	42	M	Nephrolithiasis	Aortic/Mitral/Pulmonic Valve	N	-	-	Y	N
54	92	M	Valve regurgitation	Mitral/Tricuspid Valve	N	𝛃 Lactam	4wks	Y	Y
55	61	M	Indwelling Catheter	Aortic Valve	Y	𝛃 Lactam/AG	6wks	N	Y
56	58	M	Phimosis	Aortic Valve	Y	Van	6wks	Y	Y
57	43	M	UTI/Bioprosthetic valve	Aortic Valve	N	𝛃 Lactam/Van	-	N	Y
58	86	F	Aortic stenosis	Aortic Valve	N	𝛃 Lactam/AG	6wks	N	N

## Conclusions

This case of a 75-year-old male highlights the late manifestations of untreated Aerococcus urinae infective endocarditis, the importance of promptly diagnosing endocarditis with valvular involvement, and the need for immediate surgical evaluation. We have compiled an extensive review of 58 documented cases of Aerococcus urinae endocarditis, which currently accounts for all known documented cases at this time. While more studies are required to determine the optimal choice, therapy, and duration of antibiotics, the most recent 2020 American Heart Association (AHA)/American College of Cardiology (ACC) Class 1 surgical indication guidelines for the management of patients with valvular heart disease have clearly demonstrated the importance of immediate surgical evaluation even prior to the completion of antibiotic therapy. It is our hope that valvular evaluation in patients with Aerococcus bloodstream infections should be conducted immediately upon identification, as this can pave the way for surgical intervention.
